# Tibial internal rotation in combined anterior cruciate ligament and high-grade anterolateral ligament injury and its influence on ACL length

**DOI:** 10.1186/s12891-022-05218-8

**Published:** 2022-03-18

**Authors:** Sandro Hodel, Carlos Torrez, Andreas Flury, Benjamin Fritz, Matthias R. Steinwachs, Lazaros Vlachopoulos, Sandro F. Fucentese

**Affiliations:** 1 Department of Orthopedics, Balgrist University Hospital, University of Zurich, Forchstrasse 340, 8008 Zurich, Switzerland; 2 Department of Radiology, Balgrist University Hospital, University of Zurich, Forchstrasse 340, 8008 Zurich, Switzerland; 3 SportClinic Zurich Hirslanden Clinic, Witellikerstrasse, 40, 8032 Zurich, Switzerland

**Keywords:** Anterior cruciate ligament, ACL, anterolateral complex, Anterolateral ligament, Pivot shift

## Abstract

**Background:**

Assessment of combined anterolateral ligament (ALL) and anterior cruciate ligament (ACL) injury remains challenging but of high importance as the ALL is a contributing stabilizer of tibial internal rotation. The effect of preoperative static tibial internal rotation on ACL -length remains unknown. The aim of the study was analyze the effect of tibial internal rotation on ACL length in single-bundle ACL reconstructions and to quantify tibial internal rotation in combined ACL and ALL injuries.

**Methods:**

The effect of tibial internal rotation on ACL length was computed in a three-dimensional (3D) model of 10 healthy knees with 5° increments of tibial internal rotation from 0 to 30° resulting in 70 simulations. For each step ACL length was measured. ALL injury severity was graded by a blinded musculoskeletal radiologist in a retrospective analysis of 61 patients who underwent single-bundle ACL reconstruction. Preoperative tibial internal rotation was measured in magnetic resonance imaging (MRI) and its diagnostic performance was analyzed.

**Results:**

ACL length linearly increased 0.7 ± 0.1 mm (2.1 ± 0.5% of initial length) per 5° of tibial internal rotation from 0 to 30° in each patient.

Seventeen patients (27.9%) had an intact ALL (grade 0), 10 (16.4%) a grade 1, 21 (34.4%) a grade 2 and 13 (21.3%) a grade 3 injury of the ALL. Patients with a combined ACL and ALL injury grade 3 had a median static tibial internal rotation of 8.8° (interquartile range (IQR): 8.3) compared to 5.6° (IQR: 6.6) in patients with an ALL injury (grade 0–2) (*p* = 0.03). A cut-off > 13.3° of tibial internal rotation predicted a high-grade ALL injury with a specificity of 92%, a sensitivity of 30%; area under the curve (AUC) 0.70 (95% CI: 0.54–0.85) (*p* = 0.03) and an accuracy of 79%.

**Conclusion:**

ACL length linearly increases with tibial internal rotation from 0 to 30°. A combined ACL and high-grade ALL injury was associated with greater preoperative tibial internal rotation. This potentially contributes to unintentional graft laxity in ACL reconstructed patients, in particular with concomitant high-grade ALL tears.

**Study design:**

Cohort study; Level of evidence, 3.

**Supplementary Information:**

The online version contains supplementary material available at 10.1186/s12891-022-05218-8.

## Introduction

Anterior cruciate ligament (ACL) injury is one of the major sports-related injuries in young athletes [1]. Despite recent advancements in ACL-reconstruction, persistent instability remains an unsolved problem and is reported in up to 30% [2]. Consequences are decreased return to sport rates [3] and impaired knee-related quality of life despite ACL reconstruction [4]. While a majority of athletes return to their preoperative activities after ACL reconstruction [5], only 55% of professional athletes reach their pre-injury levels and even lower rates have been reported after revision ACL reconstruction [2]. Persisting rotational laxity and impaired functional outcome after ACL reconstruction might be caused by concomitant injury to the anterolateral ligament (ALL) [6–9], as previous studies have described an important role of the ALL in controlling tibial internal rotation in ACL deficient knees [10, 11].

Therefore, the indications for a combined ACL/ALL reconstruction or lateral tenodesis have been extended recently, and comprise patients younger than 20 years active in pivoting sports, excessive rotatory laxity, excessive anterior tibial translation (ATT),revision cases, ligament laxity or the presence of a Segond’s fracture [10, 12, 13]. The clinical and radiological assessment of a combined ACL and ALL injury remains challenging but crucial for optimal patient selection for concomitant ALL reconstruction, as positive impacts on the functional outcome have been demonstrated [10, 14].

**Fig. 1:**
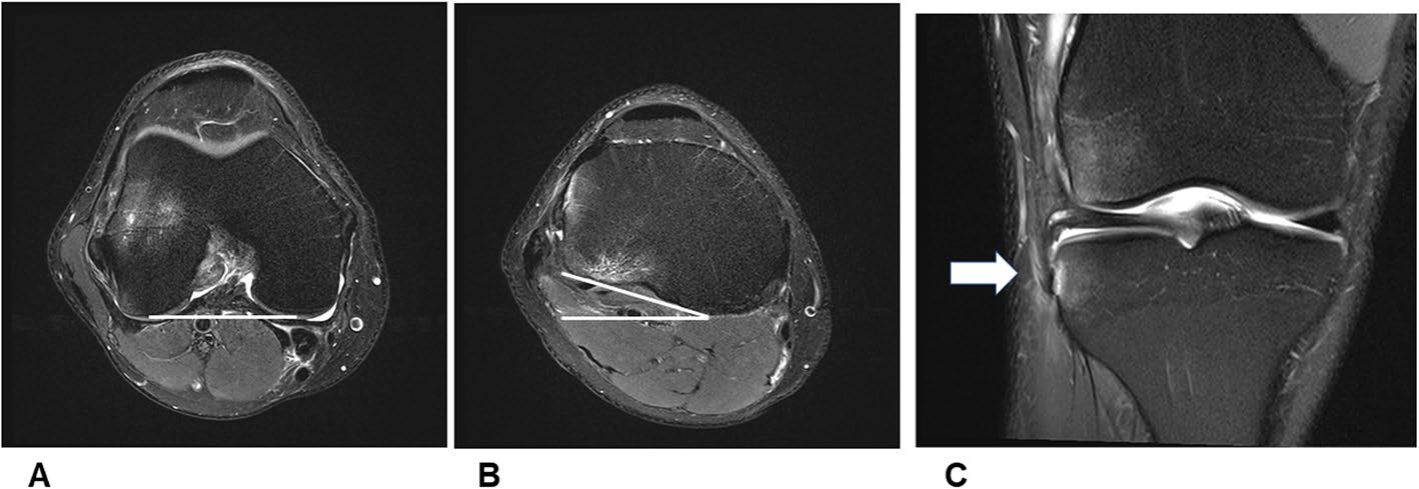
Example of a high-grade anterolateral ligament injury and tibial internal rotation in a 29-year old male. White line depicts tibial internal rotation (**B**) of 17° relative to the femoral posterior condyle line (**A**). **C** depicts the associated Segond’s fracture (white arrow

Evaluation of ALL injury by ultrasound or magnetic resonance imaging (MRI) is reported with limited inter-observer reliability [15–18]. MRI allows the detection of contour irregularity, or edema, or a complete disruption of the ALL [19]. Muramatsu et al. proposed a three-dimensional protocol and reported a higher rate of ALL injuries compared to standard protocols [20]. In addition, Monaco et al. could demonstrate a good diagnostic performance when compared to surgical exploration of the ALL in acute settings [21]. Therefore, associated injury patterns or quantitative MRI measurements could improve the diagnostic performance, and are highly desirable. Concomitant injuries in combination with an ACL and ALL injury have been described previously [22] and increased tibial internal rotation has been demonstrated in ACL deficient knees [23]. However, the extent of tibial internal rotation with respect to the ALL injury severity has not been studied yet to our knowledge (Fig. 1). Quantifying ACL lengthening due to increasing tibial internal rotation would aid to prevent undesired graft laxity.

First, we hypothesized that tibial internal rotation increases ACL length due to the excursion of the tibial ACL insertion site in the axial plane. Second, we hypothesized that increased static tibial internal rotation would be present in preoperative MRI in combined ACL and high-grade ALL injured patients compared to low-grade ALL injured patients due to the missing ligamentous restraint of tibial internal rotation and anterior tibial translation (ATT) of the lateral plateau. Therefore, the aim of the study was to compute the relationship of tibial internal rotation and ACL length in single-bundle ACL reconstruction and to assess tibial internal rotation and ATT in combined ACL and ALL injury and analyze its diagnostic reliability to detect a high-grade ALL injury.

## Methods

This study consists of two parts. In the first part we quantified the influence of tibial internal rotation on ACL length in a 3D simulation model of 10 healthy subjects.

In the second part we analyzed static tibial internal rotation in patients with a combined ACL and high-grade ALL injury.

### Part 1: the effect of tibial internal rotation on length in anterior cruciate ligament reconstruction

To compute the influence of tibial internal rotation on ACL length, 3D models of ten healthy knees (five males, five females) with a neutral leg axis (mean 0.7° varus; range 2° valgus to 4° varus) and a mean age of 24 years (range: 20 to 29 years) were generated from computer-tomography (CT) obtained for an ongoing trial. 3D triangular surface models were computed with manual threshold segmentation and region growing using MIMICS software (MIMICS, Materialize, Belgium). Afterwards, the models were imported into the in house developed surgical planning software CASPA (Balgrist, Zurich, Switzerland).

The intra-articular tibial and femoral ACL insertion points were defined based on weighted means of anatomic insertion sites as described by Parkar et al. [24] who reported the tibial insertions according to Stäubli et al. [25] and the femoral insertions according to Bernard et al. [26], as described in a previous study [27].

**Fig. 2:**
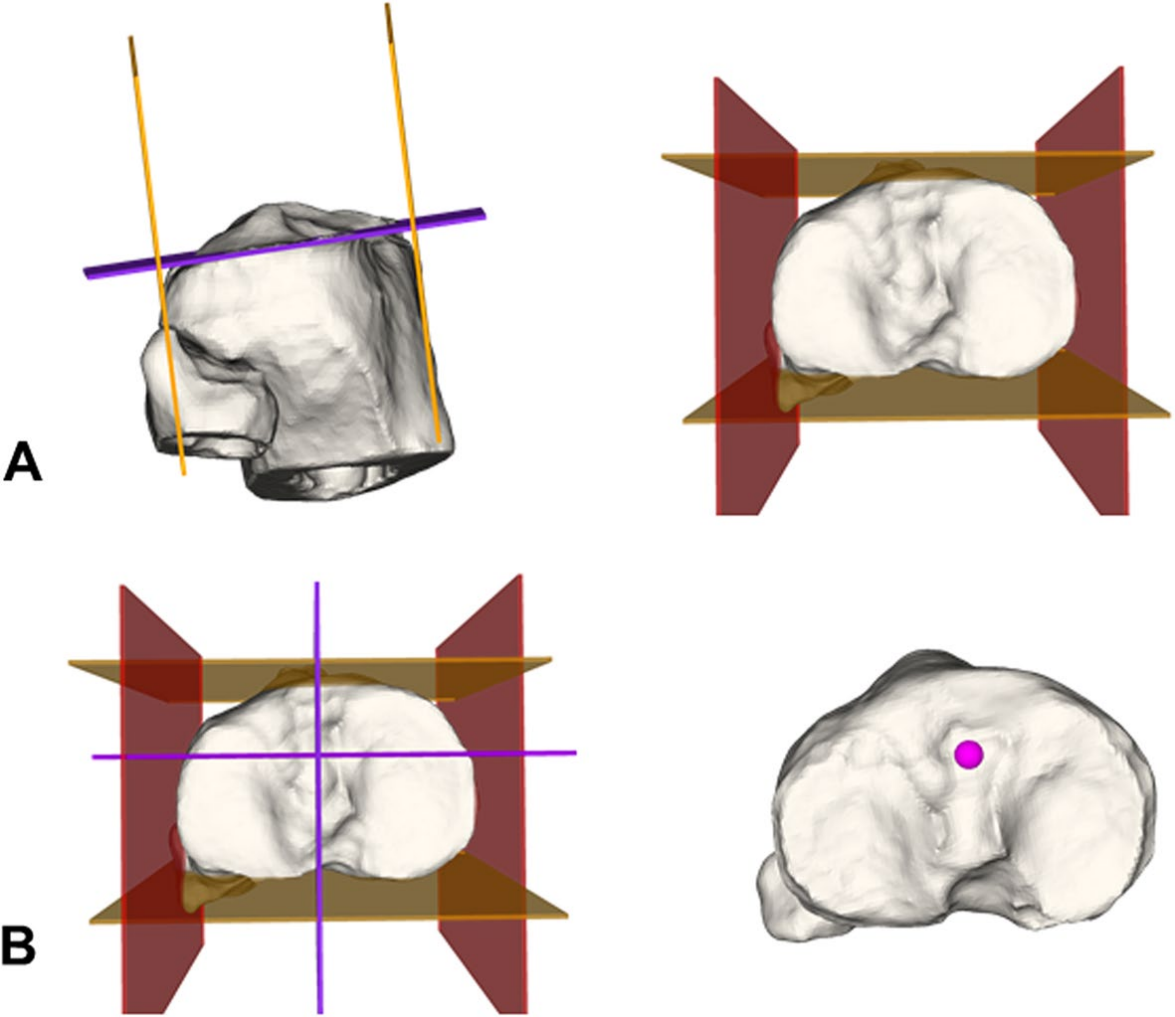
Tibial anterior cruciate ligament insertion. Definition of 3D tibial ACL insertion based on Stäubli et al. [25] and described in the text

Definition of tibial ACL insertion (Fig. 2):

1. After definition of the the tibia joint plane by five surface points on the medial and lateral plateau (violet), anterior and posterior border planes (orange) were defined to be tangent to the most anterior and posterior margin of the tibial plateau (normal vector being the cross product of the normal vector of the tibia joint plane and the tangent vector to the posterior condyles). Medial and lateral border planes (red) were defined analogue, (perpendicular to the anterior, posterior border plane and the tibial joint plane) (A).
2. The anterior border was shifted 42.3% of the total antero-posterior distance posteriorly and the medial border plane was shifted 50% of the mediolateral distance medially. The intersection (violet) defined the tibial ACL insertion (pink) (B).

**Fig. 3:**
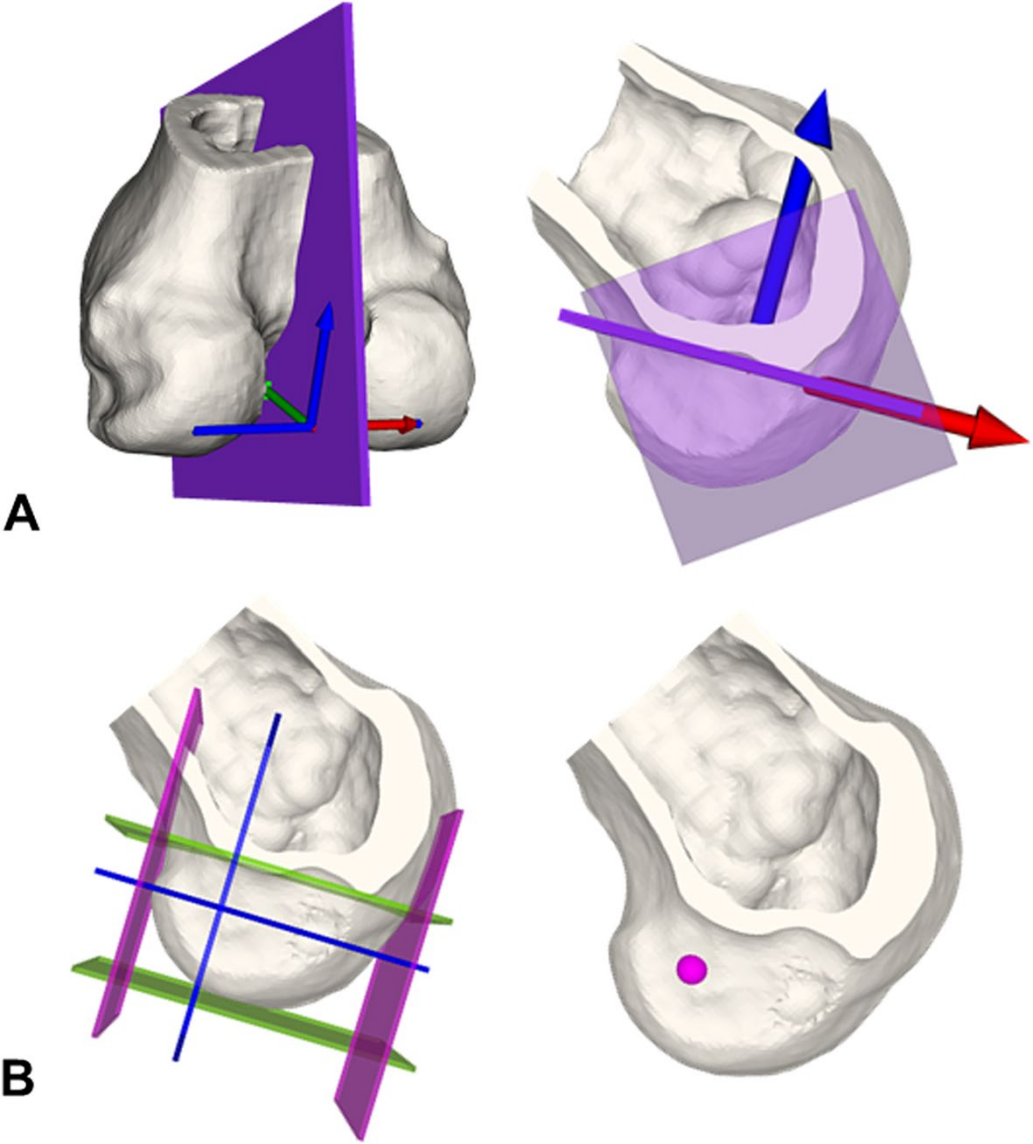
Femoral anterior cruciate ligament insertion. Definition of 3D femoral ACL insertion based on Bernard et al. [26] and described in the text

Definition of femoral ACL insertion (Fig. 3):

1. The vector between the most prominent points on the posterior medial and lateral femoral condyle defined the normal vector of a sagittal cut plane (violet) to visualize the Blumensaat’s line (A).
2. High and low border planes (green) are tangent to the Blumensaat’s line and the posterior margin of the femoral condyle (normal vector is the cross product of the normal vector of the sagittal cut plane and the tangent vector to the Blumensaat’s line). Deep and shallow border plane (pink) were defined analogue (perpendicular to the anterior/posterior border plane and the sagittal cut plane) (B).
3. The deep plane was shifted 28.6% in direction shallow and the high plane was shifted 34.5% in direction low (blue intersection) (E) and defined the femoral ACL insertion site (pink) (B).

**Fig. 4:**
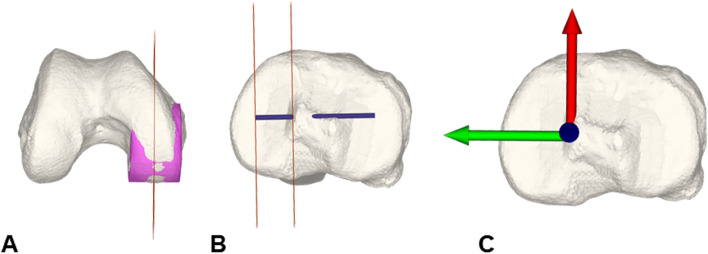
Axial center of rotation and simulation of tibial internal rotation. Axial center of rotation: 15 mm lateral to the center of the medial condyle (orange planes) on the projected femoral flexion circle axis (blue) (**A**, **B**). Tibial internal rotation was simulated around the axial center of rotation (**C**)

The axial center of rotation was computed according to Hill et al. [28] who described its location in an unloaded knee 15 mm lateral to the center of the medial condyle (A) on the axis of the flexion circle of the medial and lateral femoral condyles projected onto the tibia plateau (B). The center of rotation has the same normal vector as the tibia joint plane (Fig. 4).

While the femur and femoral ACL insertion were left unchanged, the tibia was rotated around the axial center of rotation in 5° increments starting from a neutral position (0, 5, 10, 15, 20, 25, 30 °) to a tibial internal rotation of 30° resulting in 70 simulations. For each step ACL length was measured.

### Part 2: Tibial internal rotation in combined ACL and ALL injury

A retrospective review was conducted of 61 patients who underwent single-bundle hamstring autograft ACL reconstruction at Balgrist University Hospital, Zurich, Switzerland, from 2017 to 2021. Inclusion criteria comprised a complete standard preoperative MRI assessment according to in-house protocol and complete data available and age > 18 and < 45 years. Exclusion criteria were incomplete data, multiple ligament reconstruction, previous surgery or symptoms in the affected knee, MRI findings of a partial ACL tear with a negative Lachman’s test, concurrent posterior cruciate ligament injury, posterolateral corner injury, grade > 1 medial collateral ligament (MCL) or lateral collateral ligament (LCL) injury, chronic instability, advanced articular degeneration (Outerbridge >grade 2) [29]. The sample size is the result of an a priori conducted power analysis to ensure a comparison of patients with a combined ACL and high-grade ALL injury (grade 3) to patients with an ACL and a low-grade ALL injury (grade 0–2) (see statistics). Preoperative MRI scans were analyzed on a picture archiving and communicating system (PACS) and reviewed for the presence of an ALL injury by a fellowship-trained musculoskeletal radiologist who was blinded to the study aim. MRI (1.5 Tesla or 3 Tesla) consisted of fluid sensitive fat suppressed and non-fat suppressed MR sequences in three orthogonal planes including sagittal and axial cartilage sensitive sequences with 3 mm slice thickness. MRI scans were performed supine and all legs were positioned in the MRI coil in extension. ALL injury was graded at the time of injury as: intact (grade 0), strained (grade 1), partial (grade 2) (grade 0–2: further referred to as low-grade) and complete (grade 3: further referred to as high-grade) according to van Dyck et al. [19]. An osseous avulsion of the ALL (Segond’s fracture) [30] was also classified as a grade 3 ALL injury. The location and extent of additional ligament, meniscal and cartilage injuries was assessed and arthroscopically confirmed. Tibial internal rotation was assessed as described by Vassalou et al. [23] and ATT of the medial and lateral plateau were measured preoperatively according to Tanaka et al. [31]. For inter-reader reliability, tibial internal rotation was measured by two independent readers. For intra-reader reliability, one reader repeated the measurements in 30 randomly selected patients 2 weeks later.

### Statistics

For part two a sample size calculation was performed with significance level set: α = 0.05, power level β = 0.80, assuming a tibial rotation internal rotation of 10.7° + − 4.8 of ACL injured patients [23], adequately powered to detect a minimum increase of 5° of tibial internal rotation in patients with a combined ACL and high-grade ALL injury with an estimated incidence of high-grade ALL injury of 20% [32]. This resulted in a sample size of 56 comprising 9 high-grade and 47 low-grade ALL injuries, which could be achieved with the current study population. Power analysis was performed using G*Power (version 3.1; Franz Faull, Universität Kiel) (see supplementary material).

Inter- and intra-reader reliability was calculated using intraclass correlation coefficients (ICC) (ICC model: two-way mixed-effect, absolute agreement). Mean values of both readers were used for further analysis.

Normal distribution was assessed with Shapiro Wilk’s test. Median and interquartile ranges (IQR) are given for mainly non-normally distributed data. Differences between high-grade ALL injury and low-grade ALL injury were assessed with Mann-Whitney-U test (metric) and Chi-square or Fisher’s exact test (categorical). Diagnostic performance of tibial internal rotation to predict a concomitant high-grade ALL injury was computed using receiver-operating curve (ROC), area under the curve (AUC), cut-off, sensitivity, specificity and accuracy. A logistic regression model accounted for the potential confounders age, sex, body-mass index (BMI) and injury mechanism. Additionally, for part two Pearson’s correlation was used to analyze the relationship of ATT and tibial internal rotation.

The significance was set <.05. Data were analyzed with SPSS version 23 (SPSS Inc., Chicago, IL, USA).

## Results

### Part 1: the effect of tibial internal rotation on ACL length

**Fig. 5:**
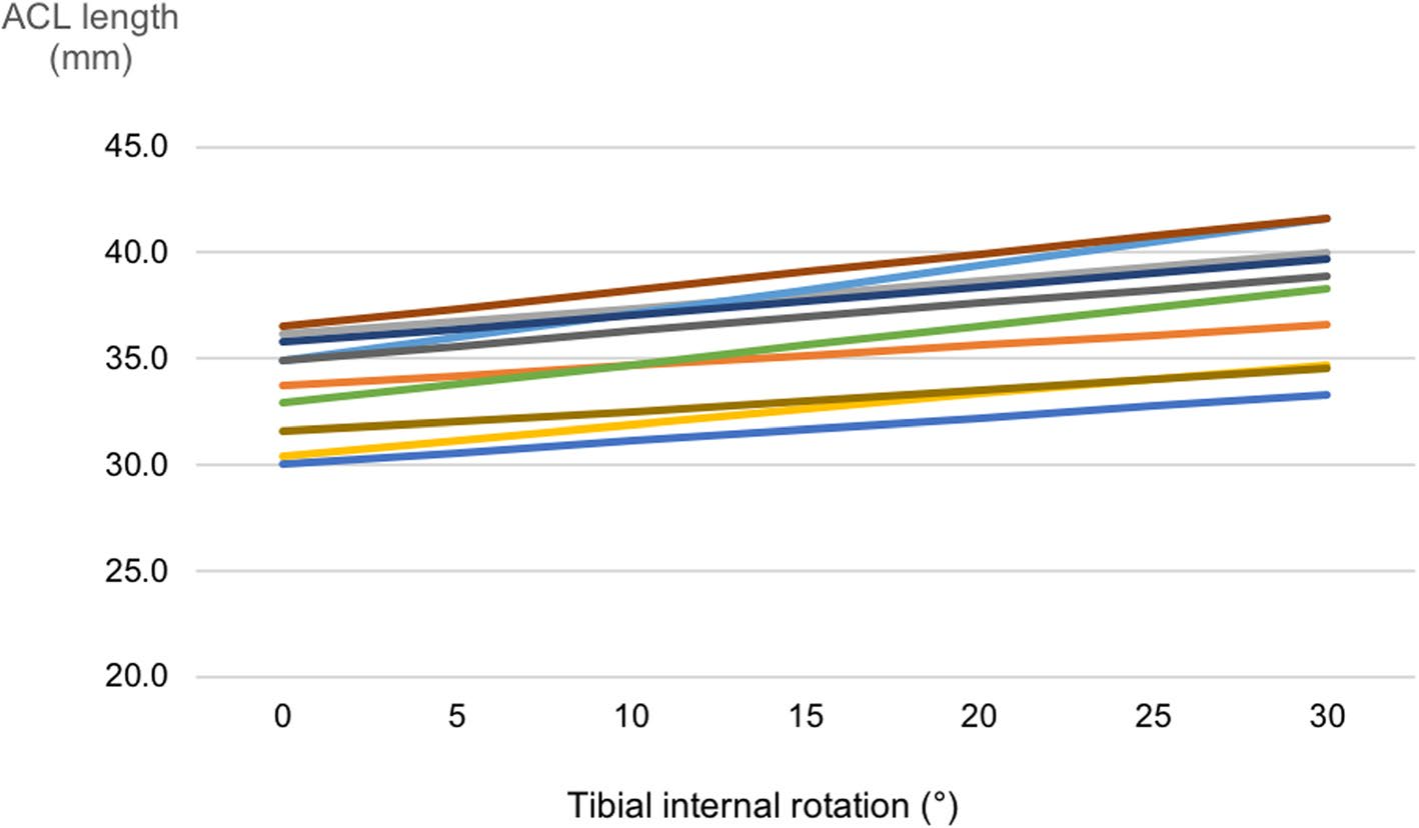
Influence of tibial internal rotation on ACL length for each knee. ACL: anterior cruciate ligament. ACL length increased linearly 0.7 ± 0.1 mm (2.1 ± 0.5% of initial length) per 5° of tibial internal rotation

Mean ACL length increased from 33.7 ± 2.2 mm (at neutral position) to 37.9 ± 2.9 mm at 30° tibial internal rotation. ACL length increased 0.7 ± 0.1 mm (2.1 ± 0.5% of initial length) per 5° of tibial internal rotation from 0 to 30° in each patient (Fig. 5).

### Part 2: Tibial internal rotation in combined ALL and ACL injury

The ICC for tibial internal rotation was 0.94 (95% CI, 0.90–0.97) for the inter-reader reliability and 0.98 (95% CI, 0.96–0.99) for the intra-reader reliability.

### Patient demographics

The 61 included patients showed no significant demographic differences and injury mechanism between ALL grades except an increased BMI in the high-grade ALL group (*p* = 0.03) (Table 1).

**Table 1:** Demographics and injury mechanism among groups

	Low-grade ALL *n* = 48	High-grade ALL *n* = 13	***p***-value
Age (years)	27 (12.5)	30 (11.5)	0.11
Male	28 (58.3%)	11 (84.6%)	0.11
Female	20 (41.7%)	2 (15.4%)	
BMI (kg/m^2)^	23.6 (3.5)	26.0 (4.1)	**0.03**
Non-contact injury	43 (89.6%)	11 (84.6%)	0.63

Numerical values reported as median (IQR), categorical variables reported as counts (%). *ALL* Anterolateral ligament, *BMI* Body mass index. Categorical values: Fisher’s exact test, metric values: Mann Whitney U test: Significant *p*-values marked bold

### Test reliability

**Fig. 6:**
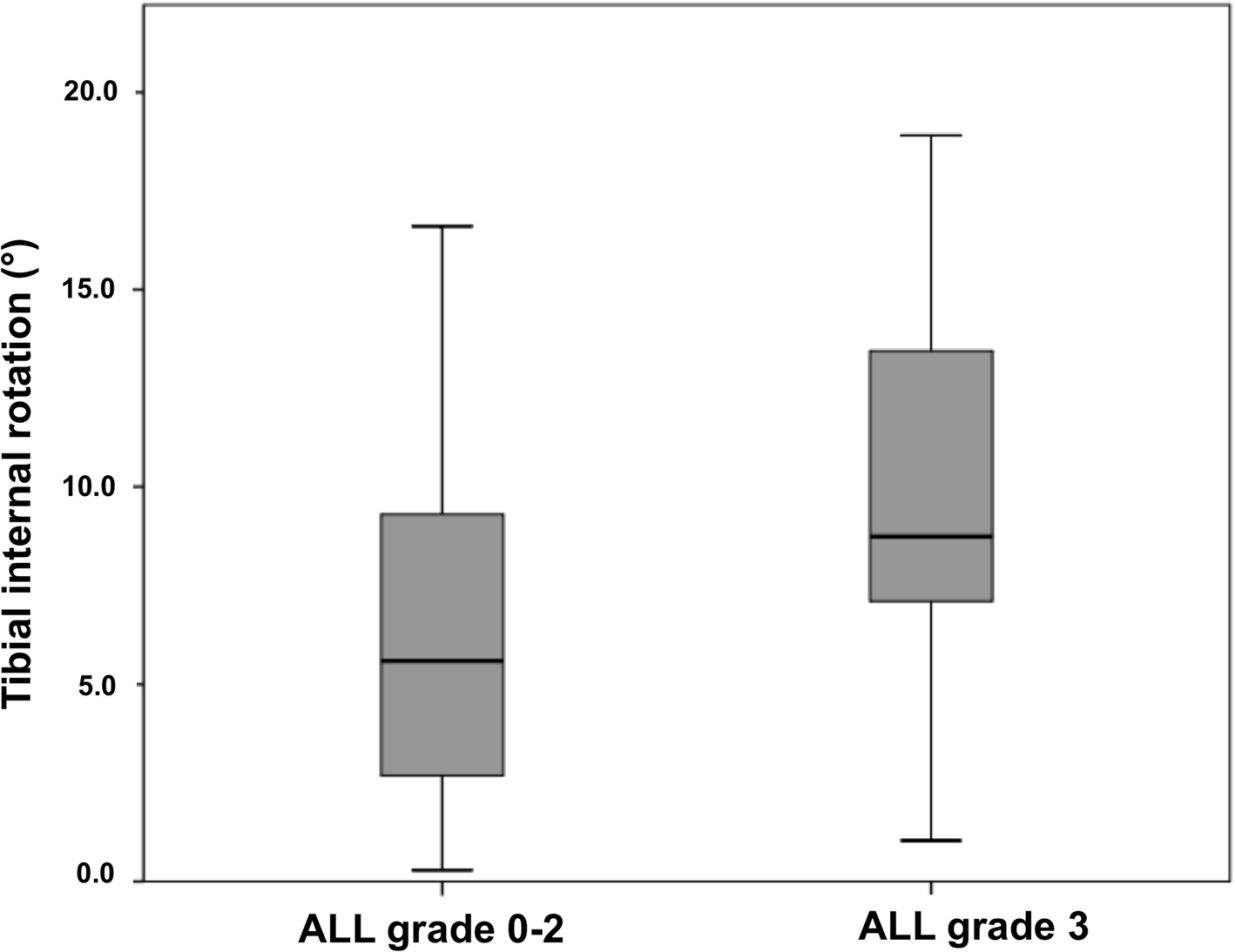
Tibial internal rotation according to anterolateral ligament injury grade. ALL: anterolateral ligament. Boxplots depicts median (line), IQR (box), minimum and maximum (whisker). Significant higher tibial internal rotation in high-grade ALL injury compared to low-grade ALL injury (*p* = 0.03)

Seventeen patients (27.9%) had an intact ALL (grade 0), 10 (16.4%) a grade 1, 21 (34.4%) a grade 2 and 13 (21.3%) a grade 3 injury of the ALL. Patients with a combined ACL and high-grade ALL injury had a median tibial internal rotation of 8.8° (IQR: 8.3) compared to 5.6° (IQR: 6.6) in patients with a low-grade ALL injury (*p* = 0.03) (Fig. 6). A cut-off > 13.3° of tibial internal rotation predicted a high-grade ALL injury with a specificity of 92%, a sensitivity of 30%; AUC 0.70 (95% CI: 0.54–0.85; *p* = 0.03) and an accuracy of 79%. The logistic regression model revealed no significance for age, sex, BMI or injury mechanism. Associated meniscal and ligament injuries are summarized in Table 2.

**Table 2:** Associated injuries

	Low-grade ALL *n* = 48	High-grade ALL *n* = 13	***p***-value
Meniscal injury			
- lateral	8 (16.7%)	1 (7.7%)	**0.003**
- medial	8 (16.7%)	2 (14.4%)	
- combined (medial and lateral)	1 (2.1%)	5 (38.5%)	
Collateral ligament injury			0.54
- LCL (grade 1)	2 (4.2%)	3 (23.1%)	
- MCL (grade 1)	4 (8.4%)	1 (7.7%)	

Numerical values reported as median (IQR), categorical variables reported as counts (%). *ALL* Anterolateral complex, *ATT* Anterior tibial translation, *LCL* Lateral collateral ligament, *MCL* Medial collateral ligament. Categorical values: Fisher’s exact test, numerical values: Mann Whitney U test. Significant *p*-values marked bold

Tibial internal rotation correlated with ATT of the lateral compartment (*r* = 0.60, *p* = 0.001). There was no significant difference of tibial internal rotation among males and females (*p* = 0.095).

## Discussion

The most important finding of this study is that an increased static tibial internal rotation indicates a high-grade ALL injury in ACL injured patients. A linear relationship of ACL length and tibial internal rotation could be established in the presented 3D model from 0 to 30°. These findings aid in identifying patients with a high-grade ALL injury and prevent ACL fixation in internal rotation and subsequent laxity.

The ALL complex has been described to play a major role as a secondary restraint of tibial internal rotation in ACL deficient knees [6, 33, 34] but also the lateral meniscus is considered a critical stabilizer of tibial internal rotation. Despite the absence of posterolateral meniscus root (PLMR) tears in our cohort, the increased incidence of isolated lateral meniscus tears in the low-grade ALL injury group may have reduced the observed difference of tibial internal rotation between groups. Especially, as increased ATT of the lateral compartment has been described after lateral meniscectomy or in the presence of PLMR tears [35, 36].

Tibial internal rotation occurred mainly through an increased ATT of the lateral compartment as described by Bedi et al. [37]. The increased excursion of the lateral compartment supports the presence of a medially located axial center of rotation as reported by Hill et al. [28] and simulated in the first part of our study. From a biomechanical point of view, a significant rotatory laxity can be expected after a complete ligamentous disruption or osseous avulsion. Furthermore, a relationship between lower grade ALL injuries and tibial internal rotation could not be established.

As previous literature demonstrated improved clinical outcome after ALL reconstruction [9, 10, 13], the clinical relevance of partial ALL ruptures or strains is of high interest to define more detailed criteria for a simultaneous ALL reconstruction in the future. Moreover, the healing potential of the ALL is low and a rotatory laxity most likely will persist if treated non-operatively [38, 39].

Regarding the diagnostic performance of the MRI several factors have to be considered that explain the variability in identifying concomitant ALL injuries as: time since injury, incomplete visualization of the ALL depending on MRI protocol and slice thickness and knee positioning [40–42].

The demonstrated increase in tibial internal rotation showed a highly linear relationship to ACL length. If the ACL is fixed in a forced internally rotated subluxation of the tibia an unintentional overlength of the ACL results in increased laxity. This effect potentially contributes to residual anteroposterior and rotatory instability, impaired functional outcome and subsequent failure in combined ACL ALL injured patients [43]. Therefore, avoiding tibial internal rotation might decrease residual laxity after ACL reconstruction. The extent of which the tibia rotates internally during fixation of the ACL could not be quantified in this study but can be expected multiples of the reported static internal rotation, as biomechanical studies have shown a tibial internal rotation up to 24° in ACL and ALL deficient knees [6]. A tibial internal rotation of 30° resulted in an increase of ACL length of 4.2 mm (12.6%) and subsequently is expected to cause significant laxity. Moreover, the clinical relevance of these findings is highlighted in multiligament knee injuries, as these are at risk for increased tibial internal rotation [44].

### Limitations

There are several limitations inherent to the study design regarding the first part. Regarding the first part, the 3D model does not take ligament restraint, muscle activity or weight-bearing load into account and the center of rotation is based on previous results of healthy knees [28]. The exact axial center of rotation in patients with a combined ACL and ALL injury is not known but is likely to influence ACL length. Moreover, the optimal tensioning of the ACL remains debatable and cannot be answered based on our findings. However, the demonstrated increase of ACL length with increasing tibial internal rotation provides the base for future research investigating optimal tensioning in combined ACL and ALL injuries.

The lack of an exact case-control matching potentially introduces bias. However, this issue was addressed using a multiple logistic regression model that did not show underlying confounders for age, sex, BMI and injury mechanism. Similarly, associated injury to the meniscus or collateral ligaments poses a potential bias that has been discussed above. However, exact matching for meniscal and ligamentous injuries is hardly feasible in a retrospective single-center study design. Other confounding factors to be considered to influence tibial internal rotation are knee flexion [45] and quadriceps activity [46]. Both factors, most likely, can be neglected due to uniform positioning of the knees in the MRI coil and absence of active voluntary quadriceps muscle contraction during image acquisition. A further limitation is that the ALL grading was completed by a single-observer, however, blinded to the study aim. The relationship of tibial internal rotation to quantitative pivot measurement, functional outcome or residual laxity after ACL reconstruction should be the subject of further investigations.

## Conclusion

ACL length linearly increases with tibial internal rotation from 0 to 30°. A combined ACL and high-grade ALL injury was associated with greater preoperative tibial internal rotation. This potentially contributes to unintentional graft laxity in ACL reconstructed patients, in particular with concomitant high-grade ALL tears.

## Supplementary Information

**Additional file 1.**

## Data Availability

The datasets used and/or analyzed during the current study are available from the corresponding author on reasonable request.

## Abbreviations

3D: Three-dimensional
ACL: Anterior cruciate ligament
ALL: Anterolateral ligament
ATT: Anterior tibial translation
AUC: Area under the curve
BMI: Body-mass index
CT: Computer-tomography
ICC: Intraclass correlation coefficient
IQR: Interquartile range
LCL: Lateral collateral ligament
MCL: Medial collateral ligament
MRI: Magnet resonance imaging
PLMR: Posterolateral meniscus root
