# Supplementary material for: Tibial internal rotation in combined anterior cruciate ligament and high-grade anterolateral ligament injury and its influence on ACL length
Source: BMC Musculoskelet Disord. 2022 Mar 18;23:262. doi: 10.1186/s12891-022-05218-8 (PMC8932291; doi:10.1186/s12891-022-05218-8)

**Sample size calculation**

**t tests** - Means: Difference between two independent means (two groups)

**Analysis:** A priori: Compute required sample size

**Input:** Tail(s) = Two

Effect size d = 1.041667

α err prob = 0.05

Power (1-β err prob) = 0.8

Allocation ratio N2/N1 = 5

**Output:** Noncentrality parameter δ = 2.8628927

Critical t = 2.0048793

Df = 54

Sample size group 1 = 9

Sample size group 2 = 47

Total sample size = 56

Actual power = 0.8028030


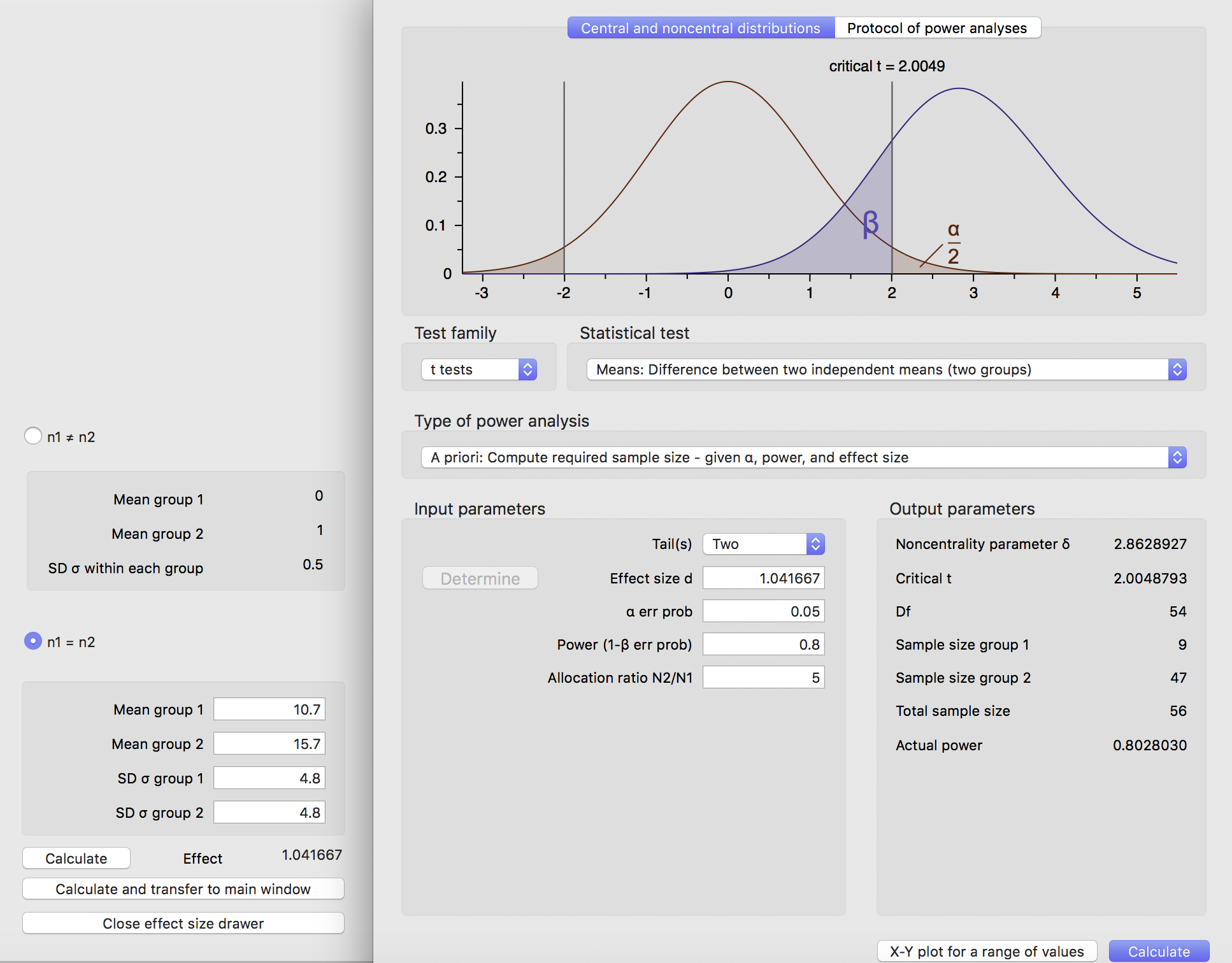

Supplement: Supplementary file 1 — Additional file 1. [file 12891_2022_5218_MOESM1_ESM.docx]
